# Macrophage Activation Associated with Chronic Murine Cytomegalovirus Infection Results in More Severe Experimental Choroidal Neovascularization

**DOI:** 10.1371/journal.ppat.1002671

**Published:** 2012-04-26

**Authors:** Scott W. Cousins, Diego G. Espinosa-Heidmann, Daniel M. Miller, Simone Pereira-Simon, Eleut P. Hernandez, Hsin Chien, Courtney Meier-Jewett, Richard D. Dix

**Affiliations:** 1 Duke University Eye Center, Duke Center for Macular Diseases, Department of Ophthalmology, Duke University, Durham, North Carolina, United States of America; 2 Bascom Palmer Eye Institute, Department of Ophthalmology, University of Miami Miller School of Medicine, Miami, Florida, United States of America; 3 Department of Biology, Viral Immunology Center, Georgia State University, Atlanta, Georgia, United States of America; 4 Department of Ophthalmology, Emory University School of Medicine, Atlanta, Georgia, United States of America; La Jolla Institute for Allergy and Immunology, United States of America

## Abstract

The neovascular (wet) form of age-related macular degeneration (AMD) leads to vision loss due to choroidal neovascularization (CNV). Since macrophages are important in CNV development, and cytomegalovirus (CMV)-specific IgG serum titers in patients with wet AMD are elevated, we hypothesized that chronic CMV infection contributes to wet AMD, possibly by pro-angiogenic macrophage activation. This hypothesis was tested using an established mouse model of experimental CNV. At 6 days, 6 weeks, or 12 weeks after infection with murine CMV (MCMV), laser-induced CNV was performed, and CNV severity was determined 4 weeks later by analysis of choroidal flatmounts. Although all MCMV-infected mice exhibited more severe CNV when compared with control mice, the most severe CNV developed in mice with chronic infection, a time when MCMV-specific gene sequences could not be detected within choroidal tissues. Splenic macrophages collected from mice with chronic MCMV infection, however, expressed significantly greater levels of TNF-α, COX-2, MMP-9, and, most significantly, VEGF transcripts by quantitative RT-PCR assay when compared to splenic macrophages from control mice. Direct MCMV infection of monolayers of IC-21 mouse macrophages confirmed significant stimulation of VEGF mRNA and VEGF protein as determined by quantitative RT-PCR assay, ELISA, and immunostaining. Stimulation of VEGF production in vivo and in vitro was sensitive to the antiviral ganciclovir. These studies suggest that chronic CMV infection may serve as a heretofore unrecognized risk factor in the pathogenesis of wet AMD. One mechanism by which chronic CMV infection might promote increased CNV severity is via stimulation of macrophages to make pro-angiogenic factors (VEGF), an outcome that requires active virus replication.

## Author Summary

Neovascular age-related macular degeneration (AMD) is the leading cause of vision loss in the elderly. Onset of AMD is due to local production of vascular endothelial growth factor (VEGF) that promotes formation of new blood vessels in the retina, thereby leading to retinal tissue destruction and blindness. Since a clinical study by us showed that AMD patients have high amounts of antibodies to human cytomegalovirus (HCMV), we postulated that infection with HCMV might be a risk factor for AMD. To investigate this possibility, mice were infected with murine cytomegalovirus (MCMV), and at various times after infection, subjected to laser treatment of the eye to induce choroidal neovascularization, an experimental model of AMD. Most severe CNV developed in mice with chronic MCMV infection, a time when MCMV gene sequences could not be detected within eye tissues. However, splenic macrophages collected from mice with chronic MCMV infection produced high levels of gene transcripts to several pro-angiogenic factors including VEGF. MCMV infection of mouse macrophages in culture also produced high amounts of VEGF. Stimulation of VEGF production in vivo and in vitro was sensitive to antiviral treatment. Chronic HCMV infection may therefore promote AMD by stimulation of VEGF production by activated macrophages.

## Introduction

Angiogenesis, the formation of blood vessels, plays a critical role in embryonic development, wound healing, and normal physiologic processes associated with natural growth and development. On the other hand, new blood vessel growth (neovascularization) contributes to a number of pathologic conditions that include atherosclerosis and tumor formation [1], [2]. The eye is also particularly sensitive to neovascularization during which abnormal blood vessel growth within retinal or choroidal tissues leads to vision loss or blindness. Sight-threatening diseases of the eye associated with abnormal neovascularization include diabetic retinopathy [3], retinopathy of prematurity [4], and age-related macular degeneration (AMD) [5].

Of these, AMD is the leading cause of severe irreversible central vision loss and legal blindness in individuals 65 years of age or older in the United States and other developed countries [6]–[9]. Since the number of elderly persons will double by 2020, AMD is expected to become a major public health problem. Two forms of AMD are recognized [5]–[10]. The non-neovascular form (also known as “dry” or “nonexudative”) represents an early form of AMD usually associated with little visual acuity loss. It is characterized by atrophic abnormalities of the retinal pigment epithelium (RPE) and drusen, small lesions at the level of the RPE that contain granular and vesicular lipid-rich material. Over time, however, this form of AMD often progresses to the neovascular (also known as “wet” or “exudative”) form of AMD that results in significant vision loss due to the appearance of choroidal neovascularization (CNV). Although the precise events that contribute to the development of AMD remain uncertain, recent studies have implicated various immunological and inflammatory mechanisms. For example, complement deposition has been demonstrated within drusen and the choriocapillaris, and several publications have demonstrated that polymorphisms in complement factor-H are associated with an increased risk of AMD [11]–[14]. Several investigators have also identified macrophages in association with drusen as well as choroidal neovascular membranes [15]–[18] suggesting a role for macrophages in the pathophysiology of both forms of AMD. In support of this hypothesis, we [19], and others [20], have shown in a mouse model of experimental CNV that depletion of macrophages significantly decreases the size and severity of lesions.

Macrophages are immune cells of monocyte origin that are classically associated with innate immune responses, particularly inflammation [21], but they may also exhibit pro-angiogenic as well as anti-angiogenic activities [22]. Thus, macrophages may exist in different activation states [23], and individuals may therefore vary in activation states as defined by expression of cytokine transcripts as well as inducible cytokine production [24]. In fact, phenotypically polarized macrophages have been broadly classified into two main groups: classically activated (M1) macrophages and alternatively activated (M2) macrophages that are further subdivided into three subtypes [25]. Moreover, M1 macrophages exhibit an anti-angiogenic phenotype, whereas M2 macrophages exhibit a pro-angiogenic phenotype [22]–[27]. It is therefore possible that individuals with macrophages of one activation state will have a relative protective effect in AMD while individuals with macrophages of another activation state will be at risk for progressive complications. This idea is supported by our observation that the presence of highly activated macrophages is associated with a 5-fold increase in risk of having wet AMD [24].

The mechanism of macrophage activation is clearly multifactorial involving genetics, systemic health cofactors, and environmental cofactors including infection [15], [28]–[30]. Infectious pathogens have been implicated in several vascular diseases, especially atherosclerosis [29]–[32]. *Chlamydia pneumoniae*, human cytomegalovirus (HCMV), and *Helicobacter pylori* all have been implicated in promoting severity of atherosclerosis and inducing complications such as myocardial infarction [31], [33], [34]. These findings prompted us to perform a seroepidemiologic study to investigate a possible association for these infectious pathogens with neovascular AMD, a study that subsequently demonstrated a significant association with high HCMV IgG serum titers [35]. This finding differed from that of Kalayoglu and coworkers [36] whose study suggested an association between chlamydia and neovascular AMD.

Given our clinical findings [35] and our long-standing interests in the immunology and pathogenesis of cytomegalovirus retinal disease [37], we sought to test the hypothesis that chronic infection with HCMV, a common β-herpesvirus that targets myeloid lineage cells that give rise to activated macrophage cell populations in tissues [38], is a heretofore unrecognized risk factor for onset and progression of neovascular AMD. This hypothesis was tested herein using an established mouse model of laser-induced CNV [39] to evaluate the effect of systemic infection by murine cytomegalovirus, a mouse β-herpesvirus whose genomic structure and cellular/tissue tropisms parallels those of HCMV [38], on the severity of CNV lesions during acute and chronic virus infection. We observed that mice with chronic MCMV infection developed more severe CNV, and that macrophages collected from chronically infected animals were activated as determined by expression of high levels of transcripts for a number of pro-inflammatory and pro-angiogenic factors, especially the pro-angiogenic cytokine vascular endothelial growth factor (VEGF). In vitro studies confirmed that MCMV infection of a mouse macrophage cell line resulted in significant upregulation of VEGF mRNA and VEGF protein production. Subsequent in vivo and in vitro studies using the antiviral ganciclovir demonstrated that increased production of VEGF by splenic macrophages collected from chronically infected mice and by MCMV-infected mouse macrophages grown in culture was ganciclovir-sensitive, findings that suggest that active virus replication is indeed required for stimulation of VEGF production by macrophages.

## Results

### Mice with chronic systemic MCMV infection develop more severe experimental CNV

**Figure 1:**
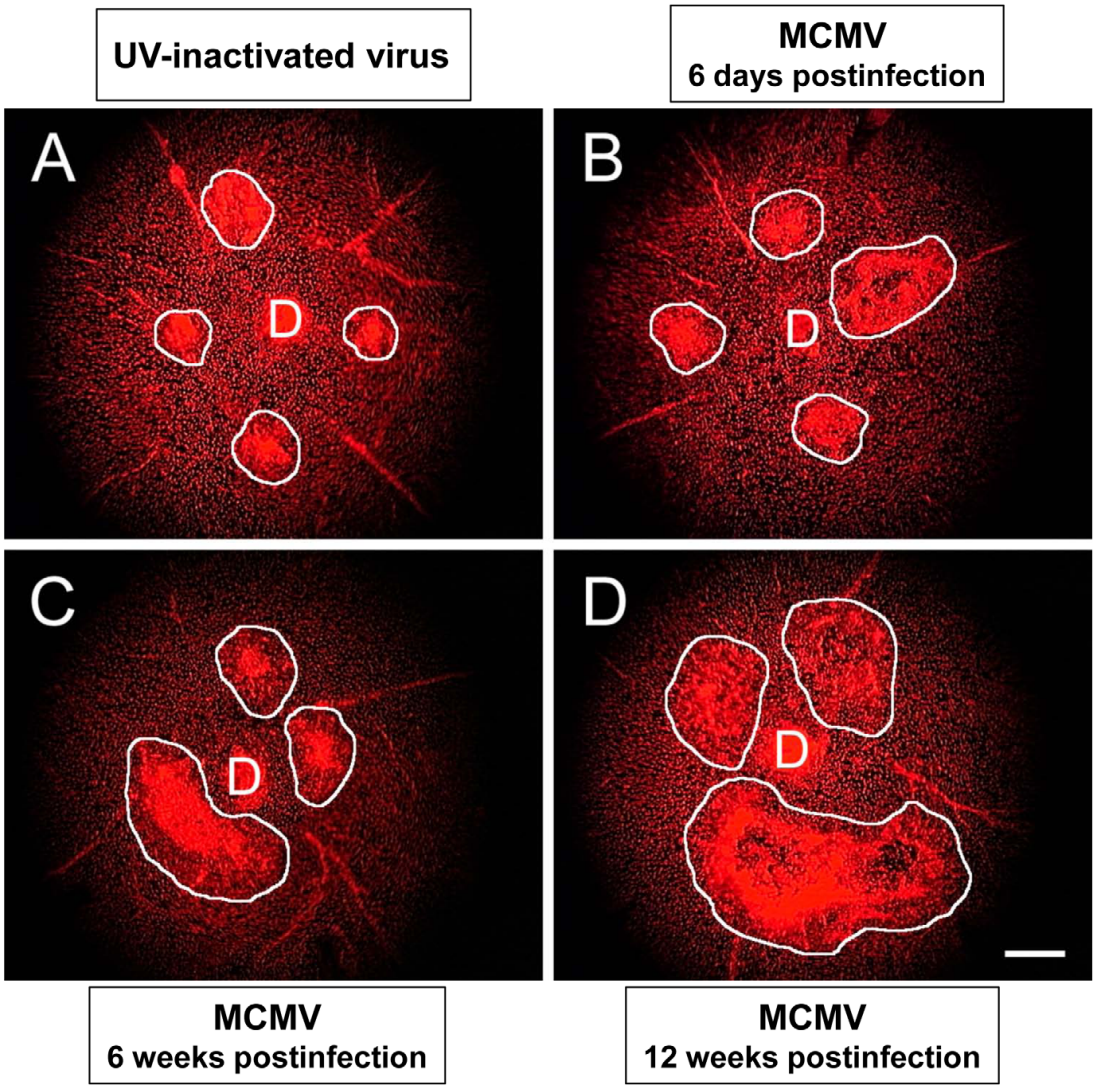
Eyes of mice with chronic MCMV infection show more severe CNV as determined by flatmount preparations. Groups of adult C57BL/6 mice were inoculated intraperitoneally with either MCMV or UV-inactivated virus. At 6 days, 6 weeks, or 12 weeks after inoculation, all mice were subjected to laser treatment to induce CNV, and, four weeks later, flatmount preparations of the posterior pole of mouse eyes were stained with propidium iodide. Flatmount preparations of representative individual mouse eyes showing areas of CNV (white outlines). (D = Optic Disc) (Magnification = 50×) (Bar = 100 um)) (**A**) Mouse inoculated with UV-inactivated MCMV (control) for 12 weeks prior to laser treatment. (**B**) Mouse infected with MCMV for 6 days prior to laser treatment. (**C**) Mouse infected with MCMV for 6 weeks prior to laser treatment. (**D**) Mouse infected with MCMV for 12 weeks prior to laser treatment.

**Figure 2:**
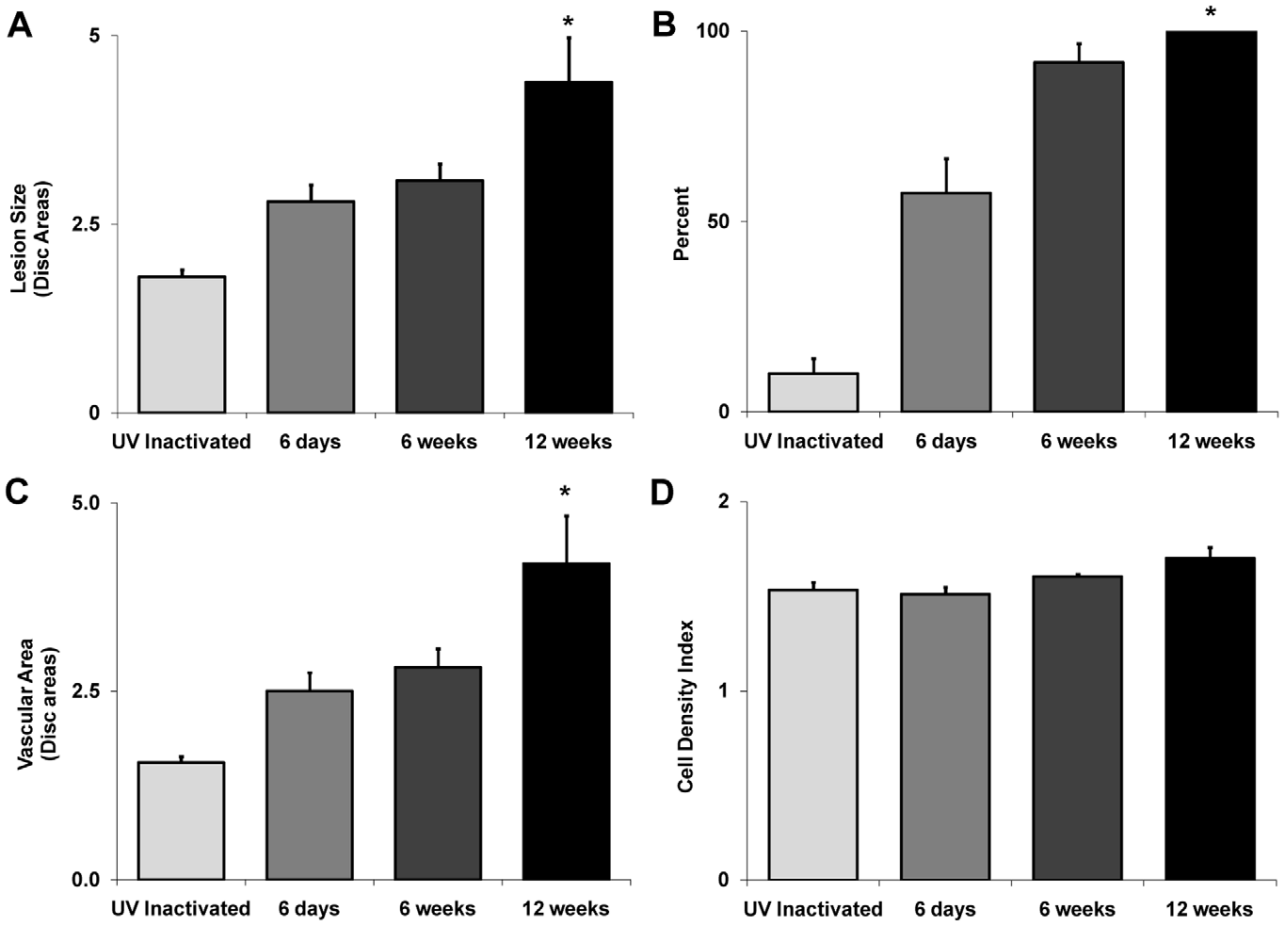
Comparative quantitative analysis of flatmount preparations of eyes of all mice of each group confirm that chronic MCMV infection results in more severe CNV. Groups of adult C57BL/6 mice were inoculated intraperitoneally with either MCMV or UV-inactivated virus (control). At 6 days, 6 weeks, or 12 weeks after inoculation, all mice were subjected to laser treatment to induce CNV, and. four weeks. later flatmount preparations of the posterior pole of mouse eyes were stained with propidium iodide and assessed for different parameters of CNV severity. Asterisks indicate statistical significance when compared with control (*p* = <0.0001). (**A**) Comparison of cellular margins (lesion size). (**B**) Comparison of frequency of large lesions (>2.2 disc areas). (**C**) Comparison of vascular area (disc areas). (**D**) Comparison of cellular density index.

We first explored the effect of systemic MCMV infection on experimental CNV. Three times relative to systemic virus inoculation were chosen for this study, one at time of acute systemic infection (6 days postinfection) and two at times of chronic systemic infection (6 weeks postinfection and 12 weeks postinfection) [38]. Groups of C57BL/6 mice were inoculated intraperitoneally with a sublethal dose of MCMV, and their eyes were subjected to laser-induced CNV [39] at 6 days, 6 weeks, or 12 weeks after systemic MCMV infection. In this study, control mice received UV-inactivated MCMV. Four weeks after laser treatment, propidium iodide-stained flatmounts of the posterior pole were prepared of all laser-treated eyes, and groups were compared for severity of CNV. Results are shown in Figure 1A–D and Figure 2A. As expected, mice inoculated with UV-inactivated MCMV exhibited small CNV lesions (1.8±0.1 disc areas). In comparison, mice inoculated with infectious MCMV exhibited CNV lesions of increased size. Lesion size also increased with time after MCMV infection. Whereas mice with MCMV infection of 6-days duration exhibited CNV lesions of moderate enlargement (2.7±0.2 disc areas) four weeks after laser treatment, progressively larger lesions were observed in mice with MCMV infection of 6-weeks (3.1±0.2 disc areas) and 12-weeks (4.4±0.6 disc areas) duration prior to laser treatment (Figure 2A). A statistical comparison of lesion sizes observed in animals with MCMV infection of 12-weeks duration versus control animals revealed significance (*p* = <0.0001). The frequency of large lesions also increased with progression of MCMV. Whereas only 10% of the total number of lesions in mice inoculated with UV-inactivated virus exceeded 2.2 disc areas (Figure 2B) (representing the 95% confidence interval for lesion size in control mice), 57.5, 92, and 100% of animals with systemic MCMV infection of 6-days, 6-weeks, and 12-weeks duration prior to laser treatment, respectively, developed large CNV lesions. Similar findings were observed when flatmounts were evaluated for degree of vascular size (Figure 2C) and vascularity (data not shown), although cellular density remained constant (Figure 2D). Taken together, these results suggest that systemic MCMV infection results in more severe CNV in mice, even during acute infection where a trend in increased severity is also observed. The most severe and statistically significant of CNV lesions, however, is found in mice with chronic MCMV infection of 12-weeks duration.

**Figure 3:**
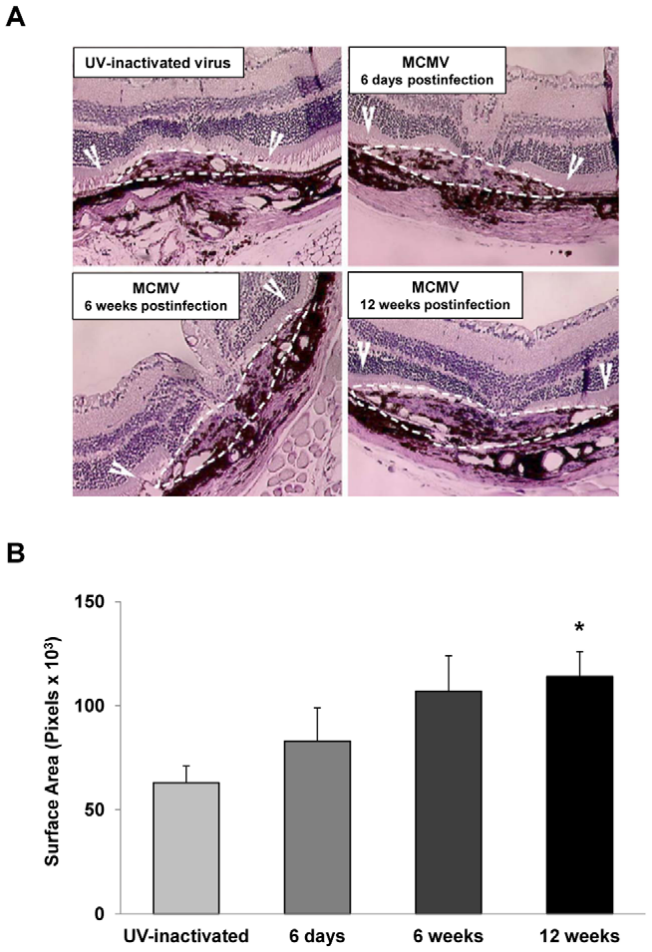
Eyes of mice with chronic MCMV infection show more severe CNV as determined by histopathologic analysis. Groups of adult C57BL/6 mice were inoculated intraperitoneally with either MCMV or UV-inactivated virus. At 6 days, 6 weeks, or 12 weeks after inoculation, all mice were subjected to laser treatment to induce CNV, and, four weeks later, retinal sections were subjected to histopathologic analysis. (**A**) Hematoxylin and eosin-stained histopathologic sections of representative individual mouse retinas four weeks after laser treatment showing areas of CNV (white outlines and arrows). (Magnification = 100×) Mouse inoculated with UV-inactivated MCMV (control); mouse infected with MCMV for 6 days prior to laser treatment; mouse infected with MCMV for 6 weeks prior to laser treatment; mouse infected with MCMV for 12 weeks prior to laser treatment. (**B**) Comparative quantitative analysis of all animals of each group for size of CNV areas as determined by histopathologic analysis. Asterisk indicates statistical significance when compared with control (*p* = <0.0004).

Histopathologic analysis of CNV lesions (Figure 3A) paralleled those of flatmount findings. When compared with mice that received UV-inactivated virus (Figure 3B), mice with laser-induced CNV at 12 weeks after infection demonstrated a near doubling of CNV surface area (64,977±7,267 pixels^2^ versus 119,149±8,578 pixels^2^; *p* = <0.0004). Importantly, neither active nor chronic systemic MCMV infection changed the typical morphological appearance of experimental CNV. There was an absolute absence of MCMV-induced cytopathology as well as retinal necrosis.

### MCMV IE1 and gH DNA cannot be detected in choroidal tissues of chronically infected mice with the most severe choroidal neovascularization

**Figure 4:**
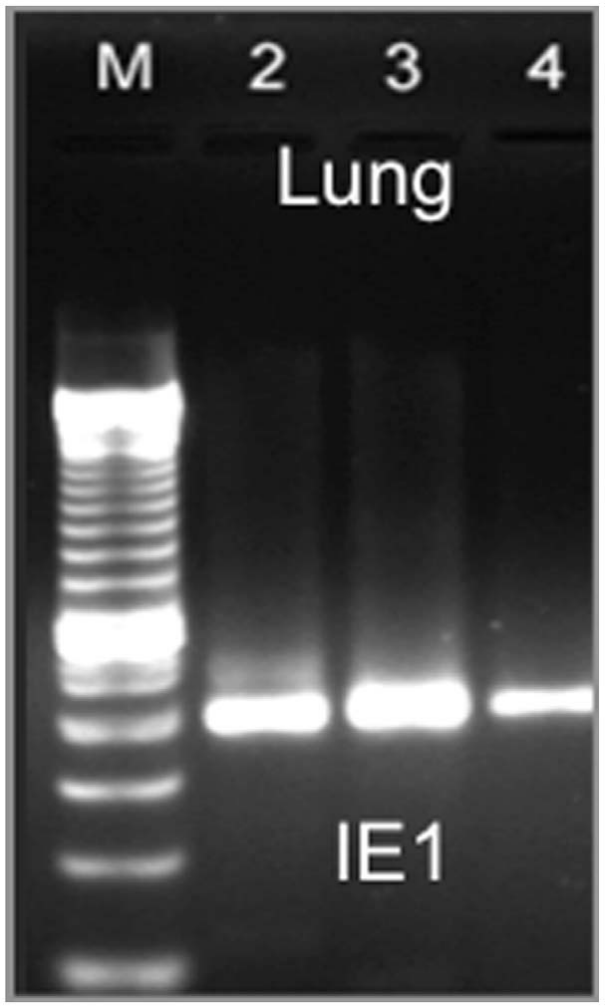
Lung tissues collected from chronically infected mice are positive for MCMV-specific IE1 DNA sequences. Representative 1% agarose gel showing positive signal for PCR assay for MCMV-specific IE1 gene sequences performed on three samples of lung tissue collected from three mice infected with MCMV for 12 weeks. (M = Molecular weight standards).

Since systemic MCMV infection was found to induce more severe CNV, we explored the possibility that direct virus infection of choroidal tissues might be responsible for this outcome. Choroidal tissues as well as several key tissues and cell populations known to be associated with MCMV pathogenesis [38], [40] were sampled for detection of MCMV DNA using primers for virus-specific immediate-early 1 (IE1) and glycoprotein H (gH) gene sequences in PCR assays. As expected, samples of spleen tissue, lung tissue (Figure 4), and salivary gland tissue as well as splenic macrophages collected from animals at time of acute MCMV infection (6 days postinfection) or chronic MCMV infection (12 weeks postinfection) provided positive signals for MCMV-specific DNA (Table 1) indicating extensive systemic MCMV infection. Purified CD34+ cells of bone marrow origin collected from mice with acute and chronic MCMV infection were also positive for MCMV DNA. In comparison, choroidal tissues from eyes of acutely infected mice were indeterminant for MCMV-specific DNA, and MCMV-specific DNA sequences could not be detected in choroidal tissues from eyes of chronically infected animals (Table 1). The apparent lack of MCMV infection of choroidal tissues taken from chronically infected animals was confirmed by our inability to recover infectious virus from whole eyes of parallel groups of chronically infected animals individually homogenized and individually inoculated onto MEF monolayers. Thus, no evidence was found for direct MCMV infection of choroidal tissues or subsequent active virus replication within the eye at time of chronic infection when CNV was found to be most severe.

**Table 1:** PCR assay analysis for MCMV-specific IE1 and gH DNA in various cells and tissues collected from mice infected with MCMV for 6 days (acute infection) or 12 weeks (chronic infection).

	Acute MCMV infection (6 days postinfection)	Chronic MCMV infection (12 weeks postinfection)
Choroidal tissue	+/−	None detected
Splenic macrophages	+	+
Whole spleen	+	+
Bone marrow cells (CD34+ cells)	+	+
Salivary gland tissue	+	+
Lung tissue	+	+

### Splenic macrophages collected from mice with chronic systemic MCMV infection are activated and produce high amounts of VEGF mRNA

Alternatively, systemic MCMV infection could contribute to increased severity of CNV indirectly via activation of macrophages to produce pro-angiogenic factors. It is well known for both HCMV and MCMV that peripheral blood monocytes are vehicles for systemic dissemination of virus during acute infection [38], [40], and these cells can harbor virus during chronic infection and with the potential to become activated macrophages within various tissues [23]. Since sufficient numbers of macrophages could not be collected from individual eyes of acutely and chronically infected mice for analysis, we subjected enriched populations of splenic F4/80+ macrophages collected from acutely and chronically infected mice to real time RT-PCR assay for detection and quantification of transcripts to several pro-inflammatory and pro-angiogenic cytokines and mediators associated with neovascular AMD. In this study, results were compared with baseline transcript levels established for splenic macrophages collected from mice inoculated with UV-inactivated virus. As shown in Table 2, significant differences were observed in the patterns of synthesis for a number of macrophage-associated transcripts examined during acute and chronic MCMV infection. Of importance was the finding of significant upregulation of VEGF (*p* = 0.04) and VEGFR1 (*p* = 0.05) transcripts that progressed from acute to chronic infection. This was associated with a concomitant significant upregulation of matrix metalloproteinase-9 (MMP-9) (*p* = 0.02), cyclooxygenase-2 (COX-2) (*p* = 0.05), and tumor necrosis factor alpha (TNF-α) (*p* = 0.03) transcripts, but only during chronic infection. Interestingly, macrophage-associated VEGFR2 transcript was significantly downregulated (*p* = 0.01) during acute and chronic infection. These results suggest that an increase in CNV size and severity during chronic MCMV infection may be due to virus-induced activation of macrophages that favor neovascularization.

**Table 2:** Macrophage mRNA levels for various cytokines and mediators relevant to CNV formation following acute or chronic MCMV infection.

mRNA levels (splenic macrophages)	UV-inactivated controls	Acute MCMV infection (6 days postinfection)	Chronic MCMV infection (12 weeks postinfection)
TNF-α	100%	77%	186% *
VEGF	100%	1,270% *	2,090% *
VEGFR1	100%	398% *	431% *
VEGFR2	100%	14% *	30% *
MMP-9	100%	32%	344% *
COX-2	100%	177%	489% *
iNOS	100%	122%	157%
PDGF-β	100%	41%	91%

Populations of splenic macrophages collected from groups of mice with acute MCMV infection (6 days postinfection) and chronic MCMV infection (12 weeks postinfection) were compared with splenic macrophages collected from groups of mice inoculated with UV-inactivated MCMV for 6 days or 12 weeks duration for mRNA levels to various pro-inflammatory and pro-angiogenic cytokines and mediators associated with neovascular AMD. These included tumor necrosis factor-alpha (TNF-α), vascular endothelial growth factor (VEGF), vascular endothelial growth factor receptor 1 (VEGFR1), vascular endothelial growth factor receptor 2 (VEGFR2), matrix metalloproteinase-9 (MMP-9), cyclooxygenase-2 (COX-2), inducible nitric oxide synthase (iNOS), and platelet-derived growth factor-beta (PDGF-β). Baseline transcript levels for splenic macrophages collected from groups of mice inoculated with UV-inactivated mice for 6 days or 12 weeks was were set at 100%. Asterisks indicate statistical significance when compared with parallel control groups.

### MCMV infection of monolayers of IC-21 mouse macrophages stimulates production of VEGF mRNA and VEGF protein

**Figure 5:**
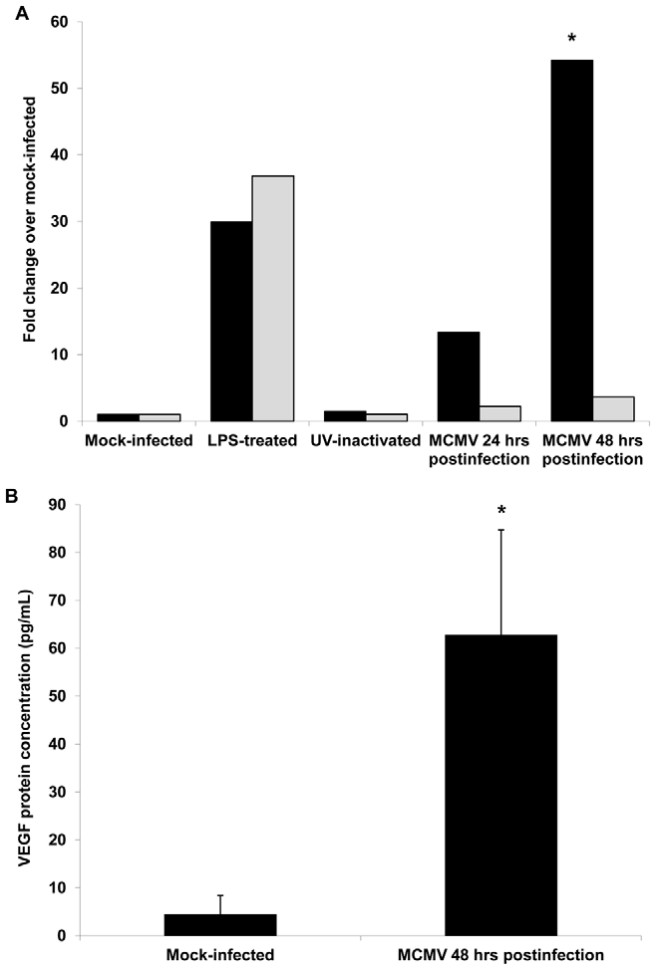
MCMV infection stimulates production of VEGF mRNA and VEGF protein in monolayers of IC-21 mouse macrophages. Monolayers of IC-21 mouse macrophages were either mock infected or infected with MCMV (2.5 PFU/cell) and analyzed for amounts of VEGF mRNA and VEGF protein at 24 hour and/or 48 hour postinfection. Asterisks indicate statistical significance when compared with mock-infected monolayers. (**A**) Quantitative RT-PCR assay for VEGF mRNA (black bars) and TNF-α mRNA (gray bars) in monolayers of IC-21 macrophages either mock-infected, treated with LPS, inoculated with UV-inactivated virus, or infected with MCMV at 24 hr or 48 hr after infection and compared with mock-infected monolayers. (*p* = ≤0.04) (**B**) ELISA for VEGF protein (black bars) in monolayers of IC-21 macrophages infected with MCMV at 48 hr postinfection and compared with mock-infected monolayers (*p* = 0.01).

Although splenic macrophages collected from mice with chronic systemic MCMV infection exhibited an approximate 20-fold increase in VEGF mRNA levels when compared with splenic macrophages collected from control mice, it is possible that increased VEGF mRNA production was not due to active MCMV replication. To explore directly the ability of mouse macrophages to produce increased amounts of VEGF during active virus replication, monolayers of IC-21 mouse macrophages, a macrophage cell line of C57BL/6 origin [41], were either mock-infected (control), treated with LPS (positive control), inoculated with UV-inactivated MCMV (negative control), or inoculated with infectious MCMV at a dose resulting in a low level of infection (2.5 PFU/cell). All monolayers were quantified at 24 hr and 48 hr later for levels of TNF-α mRNA and VEGF mRNA by quantitative RT-PCR assay. Results are shown in Figure 5A. When compared with mock-infected monolayers, monolayers of IC-21 mouse macrophages were activated by LPS treatment as demonstrated by large increases in VEGF mRNA and TNF-α mRNA levels, but parallel monolayers inoculated with UV-inactivated virus produced only low levels of VEGF mRNA and TNF-α mRNA suggesting little-to-no activation. In comparison, MCMV-infected monolayers of IC-21 mouse macrophages at 24 hr postinfection showed a 13-fold increase in VEGF mRNA levels, but interestingly failed to duplicate an increase in TNF-α mRNA production as seen in LPS-treated MCMV-infected monolayers. The same pattern of cytokine mRNA synthesis was observed in MCMV-infected IC-21 mouse macrophages at 48 hr postinfection. At this time after virus infection, VEGF mRNA levels were >50-fold greater than levels found in mock-infected monolayers (*p* = <0.04), but TNF-α mRNA levels were only ∼3-fold greater. This pattern of activation is consistent with a M2 phenotype of macrophage activation [25] since further analysis of MCMV-infected IC-21 macrophages when compared with mock-infected cells revealed increased levels of IL-10 and IL-1RA mRNA levels, equivalent levels of IL-23 mRNA production, and no detectable IL-21 mRNA production (data not shown). Confirmation that MCMV infection of IC-21 mouse macrophages resulted not only in a significant increase in VEGF mRNA levels, but also in a significant increase in VEGF protein, was provided by ELISA analysis of supernatants collected at 48 hr postinfection (*p* = 0.01) (Figure 5B). Taken together, these results provide proof-of-principal that the increase in VEGF mRNA levels observed in mice with chronic systemic infection could arise from direct MCMV infection, active virus replication, and subsequent macrophage activation associated with the M2 phenotype, a pro-angiogenic phenotype [25].

**Figure 6:**
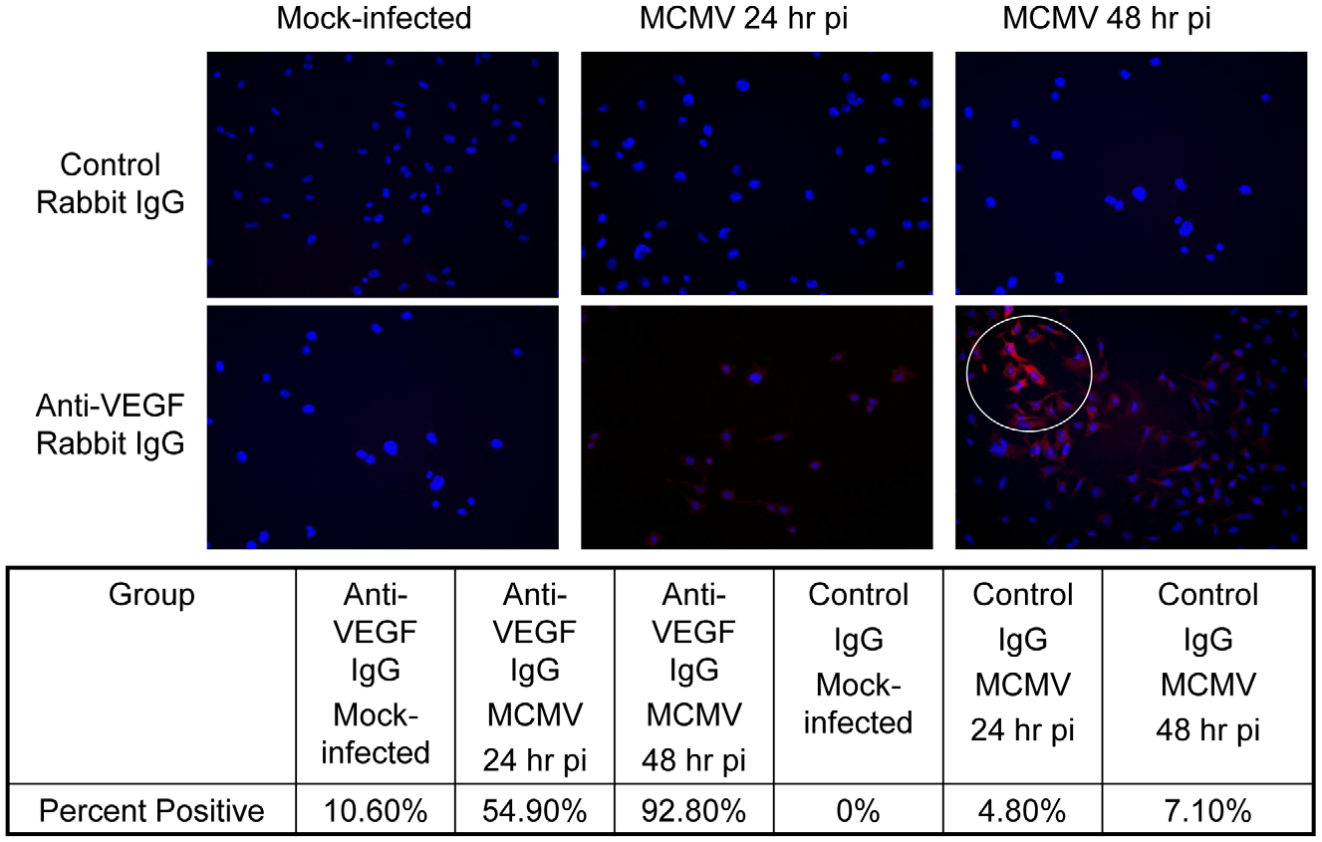
MCMV infection of monolayers of IC-21 mouse macrophages results in VEGF production by virus-infected and bystander uninfected cells. Monolayers of IC-21 mouse macrophages were either mock infected or infected with MCMV (2.5 PFU/cell). At 24 hour postinfection (MCMV 24 hr pi) or 48 hour postinfection (MCMV 48 hr pi), all mock-infected and MCMV-infected cells were harvested, fixed, and reacted with either control rabbit IgG or anti-VEGF rabbit IgG as primary antibody for visualization of VEGF production. All cells were counterstained with DAPI to visualize cell nuclei. VEGF = Red; DAPI = Blue (Magnification = 200×) White circle emphasizes focus of cells at 48 hr postinfection undergoing cytopathology and plaque formation. Quantification of cells exhibiting positive staining for VEGF was accomplished by using mean values of the number of cells showing obvious red cytoplasmic staining within five random microscopic fields of view.

To further explore VEGF production by MCMV-infected mouse macrophages in culture, monolayers of IC-21 mouse macrophages were either MCMV-infected (2.5 PFU/cell) or mock-infected and subjected to immunostaining analysis for detection of VEGF production and for quantification of VEGF-positive cells at 24 hr and 48 hr postinfection. Results are shown in Figure 6. When compared with MCMV-infected and mock-infected cells reacted with control antibody, MCMV-infected cells reacted with anti-VEGF antibody at 24 hr and 48 hr postinfection exhibited positive cytoplasmic staining for VEGF. Whereas staining was generally stronger in MCMV-infected cells at 48 hr postinfection when compared with MCMV-infected cells at 24 hr postinfection, the strongest staining was observed in foci of MCMV-infected cells at 48 hr postinfection showing early stages of cytopathology during plaque formation. It is noteworthy that individual macrophages at 48 hr postinfection not involved in plaque formation were also VEGF positive. Quantification studies revealed that ∼55% and ∼93% of MCMV-infected IC-21 mouse macrophages exhibited positive staining for VEGF at 24 hr and 48 hr postinfection, respectively, whereas mock-infected controls showed background levels of VEGF production of ∼10%.

### Production of VEGF mRNA by splenic macrophages collected from chronically infected mice and production of VEGF mRNA and VEGF protein by MCMV-infected mouse macrophages in culture is ganciclovir sensitive

**Figure 7:**
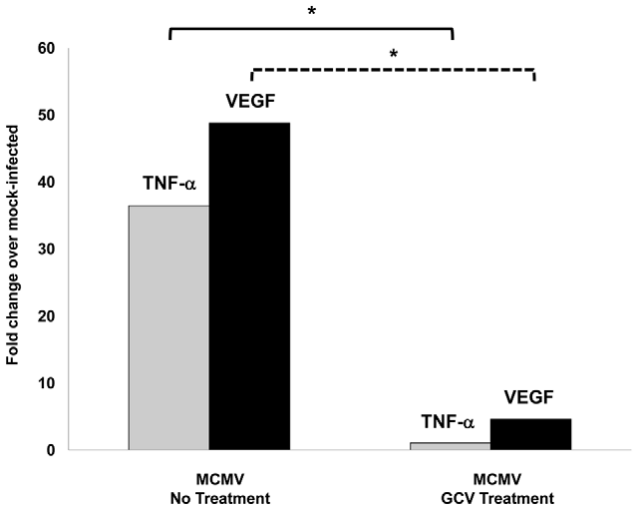
Ganciclvir treatment reduces levels of VEGF mRNA and TNF-α mRNA production by splenic macrophages collected from chronically infected mice. Groups of adult C57BL/6 mice were inoculated intraperitoneally with either MCMV or mock-infected with maintenance medium. At 12 weeks after inoculation, groups of MCMV-infected or mock-infected mice were treated intraperitoneally with ganciclovir for 7 days (40 mg/kg/day). Parallel groups of untreated control MCMV-infected or mock-infected mice received daily intraperitoneal injections of phosphate-buffered saline for 7 days. Following the 7-day regimen of ganciclovir or phosphate-buffered saline treatment, splenic macrophages were collected from treated and untreated MCMV-infected animal groups and compared with splenic macrophages collected from treated and untreated mock-infected animal groups by quantitative real-time RT-PCR assay for comparison of levels of TNF-α mRNA and VEGF mRNA production. Asterisks indicate statistical significance when compared with parallel treated or untreated mock-infected animals. (p = ≤0.009). Gray bars = TNF-α mRNA; Black bars = VEGF mRNA.

We found in studies described above that splenic macrophages collected from acutely infected mice and chronically infected mice produced significantly more VEGF mRNA when compared with splenic macrophages collected from control mice (Table 2). In addition, monolayers of MCMV-infected IC-21 mouse macrophages produced significantly more VEGF mRNA and VEGF protein when compared with monolayers of mock-infected cells (Figures 5–6). If stimulation of VEGF production in vivo and in vitro is induced directly by active virus replication, we hypothesized that stimulation of VEGF production should be sensitive to treatment with ganciclovir, an antiviral that inhibits HCMV and MCMV replication at the level of virus DNA synthesis [42], [43]. To test this hypothesis in vivo, a study was performed in which groups of C57BL/6 mice were either inoculated intraperitoneally with a sublethal dose of MCMV or mock-infected with maintenance medium (control). Unlike the study summarized in Table 2, it is noteworthy that this study did not use inoculation with UV-inactivated virus as a control. At 12 weeks postinfection, groups of MCMV-infected mice or mock-infected mice were treated intraperitoneally with ganciclovir (40 mg/kg/day) for 7 days [43]. Parallel groups of untreated control MCMV-infected mice or mock-infected mice were not treated with ganciclovir, but instead received daily intraperitoneal injections of phosphate-buffered saline for 7 days. Following the 7-day regimen of ganciclovir or phosphate-buffered saline treatment, splenic macrophages were collected from ganciclovir-treated and untreated chronically infected mice and compared by quantitative real time RT-PCR assay for levels of VEGF mRNA and TNF-α mRNA. In agreement with our previous study summarized in Table 2, splenic macrophages collected from untreated chronically infected mice showed dramatic stimulation of VEGF mRNA production as well as TNF-α mRNA production (Figure 7). In fact, the degree of stimulation for both VEGF mRNA and TNF-α mRNA production was greater than that observed in our previous study (Table 2), especially with respect to TNF-α mRNA production. This difference might be due to the different controls used in the two separate studies, UV-inactivated virus (Table 2) versus maintenance medium (Figure 7). When compared with untreated virus-infected animals, however, ganciclovir treatment resulted in a significant inhibition of VEGF mRNA production (*p* = ≤0.009), specifically an approximate 44-fold decrease in VEGF mRNA production. A similar degree of inhibition of TNF-α mRNA production was also observed in the presence of ganciclovir treatment (p = ≤0.009). Importantly, this significant inhibition of VEGF mRNA and TNF-α mRNA production in ganciclovir-treated animals could not be attributed to drug-related toxicity since splenic macrophages collected from these animals were found to be >95% viable at time of enrichment and just prior to RT-PCR assay when analyzed by the trypan blue exclusion and MTS assays (data not shown).

**Figure 8:**
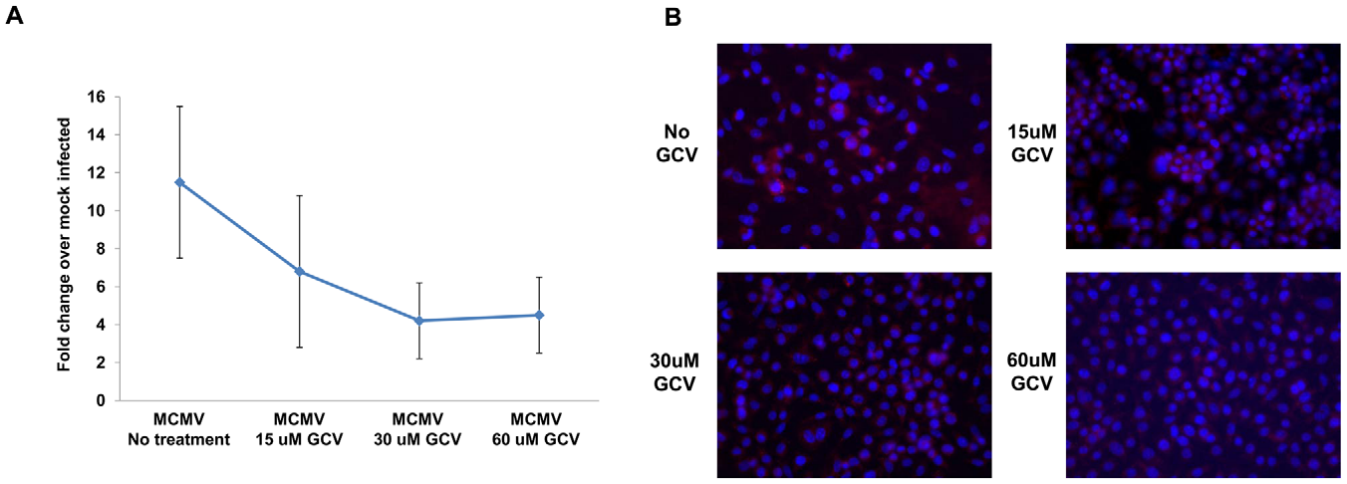
Ganciclvir treatment reduces levels of VEGF mRNA production by MCMV-infected IC-21 macrophages. Monolayers of IC-21 mouse macrophages were either mock infected or infected with MCMV (2.5 PFU/cell), treated at 1 hour postinfection with 0, 15, 30, or 60 uM of ganciclovir, and harvested at 24 hour postinfection. (**A**) Quantitative real-time PCR assay for comparison of levels of VEGF mRNA production. Three independent experiments were performed. (**B**) Immunostaining for detection of cytoplasmic VEGF production. VEGF = Red; DAPI = Blue (Magnification = 200×).

An in vitro study was performed to confirm our in vivo ganciclovir treatment findings. Monolayers of IC-21 mouse macrophages were inoculated with either a low dose of MCMV (2.5 PFU per cell) or mock-infected, and all monolayers were treated at 1 hour postinfection with either 0, 15, 30, or 60 uM of ganciclovir. At 24 hr postinfection, all monolayers were harvested and subjected to quantitative RT-PCR assay for comparison of VEGF mRNA levels. In agreement with in vivo ganciclovir treatment findings, increasing amounts of the antiviral reduced in a relatively dose-dependent manner the amounts of VEGF mRNA produced when compared with untreated MCMV-infected mouse macrophages (Figure 8A). As expected, untreated MCMV-infected mouse macrophages produced VEGF mRNA at increased levels, and at levels equivalent to that observed for MCMV-infected macrophages at 24 hr postinfection as shown in Figure 5A. With increasing doses of ganciclovir, however, amounts of VEGF mRNA were dampened, ultimately being reduced by ∼5-fold at the highest doses of ganciclovir, 30 and 60 uM. This reduction in VEGF mRNA production could not be attributed to drug-induced toxicity since mock-infected ganciclovir-treated IC-21 mouse macrophages remained >95% viable at all doses tested when subjected to the trypan blue exclusion and MTS assays (data not shown).

Since ganciclovir treatment appeared to reduce, but not eliminate, VEGF mRNA production by MCMV-infected IC-21 mouse macrophages in a relatively dose-dependent manner, we sought to determine if VEGF could be detected within ganciclovir-treated MCMV-infected IC-21 mouse macrophages, albeit at reduced levels, with increasing doses of drug. We therefore performed an immunostaining study to visualize VEGF production within monolayers of MCMV-infected IC-21 mouse macrophages at 24 hr postinfection following treatment with 0, 15, 30, or 60 uM of ganciclovir at 1 hr after virus inoculation. Results are shown in Figure 8B. In agreement with previous findings (Figure 6), MCMV-infected IC-21 mouse macrophages not treated with drug exhibited prominent cytoplasmic staining for VEGF. In comparison, increasing doses of ganciclovir treatment appeared to dampen VEGF protein production within the MCMV-infected cells. Nonetheless, positive staining for VEGF could still be detected within MCMV-infected IC-21 mouse macrophages treated with the highest dose of ganciclovir, 60 uM. Only faint background staining or no detectable staining was observed in parallel control monolayers of mouse macrophages that were either not infected with virus or virus-infected and reacted with control antibody (data not shown). Taken together, these in vitro findings suggest that VEGF mRNA and VEGF protein production during MCMV infection of IC-21 mouse macrophages are indeed ganciclovir-sensitive, although VEGF production is not completely eliminated in the presence of the antiviral. Moreover, the significant reduction of VEGF mRNA and VEGF protein during ganciclovir treatment of MCMV-infected IC-21 mouse macrophages in culture is in agreement with in vivo findings, thereby supporting the hypothesis that upregulation of VEGF mRNA within splenic macrophages collected from MCMV-infected mice with chronic infection is due to active virus replication.

## Discussion

The number of investigations of angiogenesis in the eye has increased significantly in recent years due to findings that neovascularization of the retina and choroid plays a central role in the development of a number of major blinding diseases. These include AMD as well as diabetic retinopathy, polypoidal choroidal vasculopathy, myopic choroidal neovascularization, neovascular glaucoma, retinopathy of prematurity, and ocular tumorigenesis (all reviewed in [44]). Since a seroepidemiologic clinical study by us demonstrated an apparent association between HCMV infection and neovascular AMD [35], we used an experimental C57BL/6 mouse model of CNV to test the hypothesis that systemic MCMV infection will contribute to the severity of CNV. It has not escaped our attention that mouse strain-dependent factors might play a factor in CNV development during systemic MCMV infection since macrophages from C57BL/6 mice (a prototypical Th1 mouse strain) and macrophages from BALB/c mice (a prototypical Th2 mouse strain) exhibit distinct M1- or M2-dominant responses [45]. Nonetheless, our results collectively showed that systemic MCMV infection of C57BL/6 mice did indeed result in more severe CNV, and, more importantly, chronically infected mice showed the greatest severity of CNV. Although MCMV DNA sequences could not be detected within choroidal tissues of chronically infected animals, splenic macrophages collected from chronically infected animals produced increased amounts of transcripts to several pro-inflammatory and pro-angiogenic cytokines including VEGF. That MCMV infection of mouse macrophages will modulate a pro-angiogenic M2 phenotype that included significant stimulation of VEGF production was shown directly by in vitro studies using a mouse macrophage cell line of C57BL/6 origin. Further evidence that virus infection induced stimulation of VEGF production both in vivo and in vitro was provided by ganciclovir treatment studies that demonstrated sensitivity of VEGF production to the antiviral both in vivo and in vitro. Thus, our findings are novel with respect to chronic eye disease since they provide for the first time new data that suggests that chronic cytomegalovirus infection can contribute to the pathogenesis of wet AMD, possibly via activation of macrophages towards a pro-angiogenic phenotype and stimulation of VEGF production. While we have not yet demonstrated in our model direct visualization of MCMV-infected, VEGF-producing macrophages associated with areas of CNV, several observations would argue that this is a likely occurrence. Firstly, we [19] and others [20] have shown previously that macrophages are essential for development of CNV. Secondly, we have shown previously in the context of MCMV retinitis that IC-21 macrophages infected with a β-galactsidase-expressing LacZ recombinant MCMV will travel to ocular tissues of C57BL/6 mice following tail vein injection [46]. Finally, we show herein that MCMV infection of IC-21 macrophages stimulates VEGF production, a stimulation that is also observed in splenic macrophages collected from chronically infected mice with severe CNV. Future in vivo immunostaining studies will directly address this important issue.

The concept that infectious agents might contribute to the pathogenesis of vascular diseases has become an intense and controversial area of investigation. Two major hypotheses have emerged. One hypothesis proposes that vascular disease is caused by direct infection of the target tissue [36], while the second hypothesis proposes a bystander effect caused by infection at a distant tissue [47]–[49]. In atherosclerosis, direct infection of the atheromatous plaque by *Chlamydia pneumoniae* has been suggested as a stimulus for recruitment of inflammatory cells. Arguing against this hypothesis, however, are antibiotic treatment trials designed to suppress *Chlamydia pneumoniae* infection and failing to demonstrate a measurable clinical effect on preventing myocardial infarction or other sequelae [34]. On the other hand, patients with chronic periodontal infection and inflammation have provided evidence suggesting that chronic infection at a distant site may play a role in vascular disease. In this patient population, infection by a variety of different organisms appeared to lead to more severe vascular disease [34], [50], [51]. Since in our study, MCMV-specific DNA sequences could not be detected in choroidal tissues of eyes with the most severe choroidal neovascularization, we propose a similar bystander hypothesis for the role of HCMV infection in chroroidal neovascularization of the eye.

HCMV is a common β-herpesvirus that persists for the life of its host following primary infection. While chronic HCMV infection of healthy, immunologically normal persons was initially thought to have no significant disease consequence, chronic HCMV infection has now been associated with a growing number of long-term diseases that include the vascular disease atherosclerosis, restenosis following angioplasty, transplant vascular sclerosis associated with chronic allograft rejection of solid organ grafts (reviewed in [52]), and possibly tumor formation (reviewed in [53]). Evidence for a link between HCMV and vascular disease was first provided by Melnick, DeBakey, and coworkers [54] when virus antigen was detected within arterial tissues from carotid artery plaques obtained from patients with atherosclerosis. Since this fundamental observation of ∼20 years ago, however, it has been difficult to determine the precise mechanisms by which HCMV might participate in the pathophysiology of vascular disease because the etiologies of chronic diseases are complex and multifactorial. Nonetheless, seropositive HCMV persons are two to three-times more likely to develop coronary artery disease when compared with HCMV seronegative patients [55]. In support of this association are recent findings that 76% of patients with ischemic heart disease have detectable HCMV DNA within their vascular tissues [56], and up to 53% of carotid artery atherosclerotic lesions are positive for HCMV DNA [57]. A number of animal studies have also provided compelling evidence that cytomegalovirus plays an important role in the pathophysiology of atherosclerosis, including several studies that have demonstrated more severe atherosclerosis in apoE −/− mice following systemic MCMV infection [58]–[61].

While an association has been recognized between cytomegalovirus infection and atherosclerosis, the strongest association of cytomegalovirus in vascular disease is with the development of restenosis and transplant vascular sclerosis. Several clinical studies have shown that HCMV infection is involved in accelerating both acute and chronic graft failure in all types of solid organ transplants by promoting vascular disease associated with rejection [52], probably by virus originating from the vasculature of transplanted organs from HCMV seropositive donors [62]. For example, HCMV infection was shown to double the 5-year rate of graft failure in cardiac allograft recipients due to accelerated transplant vascular sclerosis [63]. Similarly, kidney transplant allograft survival was decreased in asymptomatic HCMV-infected recipients during the first 100 days after transplantation when compared with recipient patients who had no evidence for HCMV infection, an outcome suggesting that HCMV infection, even when asymptomatic, has a negative impact on graft survival [64]. These clinical findings have been supported by a number of rat models of heart, kidney, lung, and small bowel transplantation in which infection with rat cytomegalovirus (RCMV) significantly decreased the mean time to graft failure while concomitantly increasing the degree of vasculopathy within the allograft tissue [65], [66].

Neovascularization is a complex, multi-step process of angiogenesis that rapidly takes place in response to inflammation and tissue injury, and involves many cell types, cytokines, chemokines, and proteases that work in concert to form new blood vessels from existing blood vessels. In brief (reviewed in [52]), angiogenesis is initiated by release of pro-angiogenic factors from activated endothelial cells and tissue-resident macrophages, followed by removal of pericytes that surround the existing blood vessels. This results in the breakdown of the basement membrane of the existing blood vessel wall through activation of several proteases including matrix metalloproteinases (MMPs). The release of extracellular remodeling proteins during continued degradation of the blood vessel wall leads to the release of growth factors that promote endothelial cell migration toward the angiogenic stimulus and ultimately mediates endothelial cell proliferation that drives the formation of neotubules. These neotubules in turn release additional growth factors such as platelet-derived growth factor (PDGF) that recruit vascular smooth muscle cells and pericytes that stabilize the newly formed blood vessel. Importantly, pro-angiogenic M2 macrophages have been shown recently to act as bridging cells that promote the fusion of neotubules into one continuous blood vessel [67]. Cytomegalovirus infection could therefore enhance neovascularization at various stages of angiogenesis through a number of direct and indirect mechanisms.

Monocytes are the primary target in vivo for HCMV (and MCMV and RCMV) infection [68], [69]. They serve as a site for virus latency and persistence [70], and help to disseminate virus throughout the host including the vasculature. When virus-infected monocytes enter the vasculature, they mature, and during the maturation process to become macrophages, they initiate an activation program that also serves to stimulate virus replication [71]. In this manner, infected macrophages may disseminate virus to other cells of the vasculature that are involved in angiogenesis and vascular disease. These include endothelial cells, smooth muscle cells, pericytes, and fibroblasts [52]. Given this complexity, the precise temporal relationship between virus infection of individual cell types and disease pathogenesis remains obscure and difficult to determine. Nonetheless, it is known that HCMV infection of endothelial cells induces the expression of adhesion molecules ICAM-1 and VCAM-1 [72] that serve to magnify transendothelial cell migration of inflammatory cells including monocytes. These monocytes become resident macrophages that promote angiogenesis by secretion of VEGF and other pro-angiogenic factors such as IL-6 [52].

During virus replication, the HCMV-encoded chemokine receptor US28 also plays a prominent yet multifaceted role in angiogenesis. Firstly, US28 has been shown to stimulate VEGF production directly by induction of COX-2 via activation of the NF-κB pathway [73]. Secondly, this HCMV-encoded chemokine receptor promotes the migration of macrophages in response to the CX3CL1 chemokine Fractalkine [52], a function that may help to attract additional HCMV-infected macrophages to areas of inflammation and thereby amplify angiogenesis. Thirdly, US28 also promotes the migration of vascular smooth muscle cells [74], but does so by binding to CC-chemokines and not Fractalkine [75]. Thus, US28 appears to stimulate the migration of both macrophages and vascular smooth muscle cells, but in a ligand-dependent manner. Whereas US28-induced migration of macrophages takes place after ligation with Fractalkine, but not CC-chemokines, US28-induced migration of vascular smooth muscle cells is mediated by binding to CC-chemokines, but not Fractalkine. Since HCMV-encoded US28 apparently plays multiple roles in promoting angiogenesis, we postulate the same is true for M33, the MCMV homologue of US28 [76]. Ongoing studies are therefore oriented toward testing the hypothesis that MCMV-encoded M33 plays significant roles in the pathophysiology and increased severity of CNV during chronic MCMV infection.

Additional direct and indirect mechanisms by which cytomegalovirus might contribute to angiogenesis and vascular disease are suggested by other studies. Examples include studies that have shown that HCMV infection induces a reduction of endothelial nitric oxide synthase activity commonly observed during cardiovascular disease [77]; RCMV induces the stimulation of a number of proteases including MMPs that are involved in degradation of the basement membrane required during the angiogenesis process [78]; HCMV induces an upregulation of a number of cellular chemokines including macrophage inflammatory protein 1 alpha (MIP1-α), MIP1-β, RANTES, and IL-2 that play critical roles in angiogenesis and development of vascular disease [74], [79]; and HCMV infection of coronary artery smooth muscle cells stimulates VEGF expression [80].

Since angiogenesis in health and disease is a process of great complexity that offers a number of mechanisms by which cytomegalovirus infection of multiple cell types might serve as a stimulatory cofactor in the development of more severe choroidal neovascularization, we elected to focus our study on a possible role for macrophages during chronic systemic MCMV infection. Macrophages can be either pro-angiogenic or anti-angiogenic depending on their polarization phenotype [25] that is regulated by the cytokine patterns encountered by macrophages within the resident tissue milieu [23], [26]. Classically activated macrophages, or M1 macrophages, exhibit an anti-angiogenic phenotype and produce high amounts of IL-12, IL-23, IL-6, and TNF-α, but low amounts of IL-10 [81]. In comparison, alternatively activated macrophages, or M2 macrophages, exhibit a pro-angiogenic phenotype and produce high amounts of IL-10, but low amounts of pro-inflammatory cytokines such as IL-6 and TNF-α [81]. Moreover, M1 macrophages inhibit angiogenesis by inducing a cell-death program in endothelial cells, whereas M2 macrophages promote angiogenesis by stimulating production and release of pro-angiogenic factors such as VEGF that encourage endothelial tip cell formation [52]. In this regard, Fantin and coworkers [67] have recently made the extraordinary observation that M2 macrophages may also play a critical role during formation of new blood vessels by serving as bridge cells to properly position and fuse neotubules into one continuous blood vessel, possibly via activation of the DII4-a ligand and expression of Notch receptors [82]. Thus, cytomegalovirus infection of monocytes and macrophages may influence angiogenesis-related activities by several possible mechanisms. For example, HCMV infection of monocytes appears to influence the polarization phenotype of the activated macrophage by modulating in a selective manner many M1/M2-associated factors [52], [83], thereby inducing angiogenesis through stimulation of VEGF production and other angiogenic factors. Importantly, MCMV-infected IC-21 mouse macrophages exhibited a pro-angiogenic M2 phenotype in our studies. Alternatively, HCMV infection could conceivably have a detrimental on the normal angiogenic process by promoting inflammation. HCMV infection of endothelial cells may also enhance the stability of newly formed blood vessels through stimulation and release of several cytokines and growth factors including the Notch 2 receptor [83]. We therefore postulate that chronic MCMV infection results in more severe choroidal neovascularization in our study by driving monocytes toward a M2 macrophage phenotype that favors angiogenesis through stimulation and release of pro-angiogenic factors that includes VEGF. It has not escaped our attention, however, that chronic MCMV infection might also cause more severe choroidal neovascularization by direct or indirect mechanisms associated with endothelial cell infection, a focus of future studies.

Splenic macrophages collected from chronically infected mice with the most severe choroidal neovascularization in our study showed significant increases in the amounts of transcripts to MMP-9 and COX-2, two proteins known to be involved in angiogenesis [52]. The most dramatic increase in transcript level, however, was observed for that of VEGF, a critical pro-angiogenic factor. This observation was confirmed in a second independent animal study by us that demonstrated an even greater increase in VEGF transcript production in splenic macrophages collected from chronically infected animals. One interpretation of these reproducible findings is that when chronically infected monocytes are recruited to choroidal sites of laser-induced damage, their activation programs are initiated and oriented toward the pro-angiogenic M2 phenotype. Since they are also chronically infected with MCMV, this activation program stimulates virus replication, an event that leads to enhanced production and secretion of several pro-angiogenic factors including VEGF. Inoculation of cultures of human foreskin fibroblasts or cultures of coronary artery smooth muscle cells with HCMV has been shown to result in stimulation of functionally active VEGF production [78]. It is therefore not surprising in the present study that MCMV infection of cultures of IC-21 mouse macrophages significantly stimulated production of VEGF mRNA and VEGF protein. Additional observations made during immunostaining studies also demonstrated that VEGF is indeed produced in high amounts by MCMV-infected IC-21 mouse macrophages, especially those in the early stages of cytopathology during plaque formation.

Of particular interest, however, was the additional observation that monolayer cells too early to be infected with MCMV (given the low multiplicity of infection used) were also VEGF-positive, an observation suggesting the attractive hypothesis that uninfected bystander macrophages might also be stimulated by adjacent MCMV-infected macrophages to produce enhanced amounts of VEGF during virus infection. Thus, MCMV infection of resident macrophages of tissues of the lung and spleen, and even bone marrow cells, could conceivably contribute to macrophage activation during chronic infection. MCMV-infected bone marrow cells, especially stromal cells, could favor a pro-angiogenic microenvironment that induces bystander activation during development within the marrow since stromal cells serve as a substrate upon which monocytes are induced to differentiate [84]. In addition, due to their high vascularity, both lung and spleen experience high monocyte traffic, and chronic MCMV infection of these tissues could induce bystander activation. We therefore postulate that chronic MCMV infection of monocytes and macrophages distant from the eye serves as an important mechanism for macrophage activation of the M2 phenotype that would contribute to the pro-angiogenic microenvironment of the choroidal tissues of the eye.

Treatment with ganciclovir, a potent inhibitor of active cytomegalovirus replication and HCMV disease in the clinical setting [42], [43], has been shown to delay the time to development of allograft rejection in heart transplant recipients [85], [86], a finding that underscores the importance for active HCMV replication in acceleration of vascular disease. Additional studies using experimental rat transplant models have provided similar data showing that ganciclovir therapy also reduced or prevented RCMV-associated acceleration of tissue rejection when compared with RCMV-infected animals not treated with the antiviral [87], [88]. Since we hypothesize that MCMV infection of macrophages plays a central role in amplifying the severity of experimental choroidal neovascularization in mice by stimulation of pro-angiogenic factors including VEGF, we used a similar antiviral approach to demonstrate that VEGF-specific transcript production by splenic macrophages collected from chronically infected mice was indeed ganciclovir-sensitive. This outcome strongly supports the need for active MCMV virus replication in stimulation of production of pro-angiogenic factors such as VEGF that is required for increased severity of choroidal neovascularization. These in vivo findings were duplicated and extended in culture using ganciclovir-treated, MCMV-infected monolayers of IC-21 mouse macrophages, and in a relatively dose-dependent manner.

In summary, the findings reported herein using an experimental mouse model of CNV serve to clarify our previous seroepidemiologic clinical study in which a significant association was identified between high titers of anti-HCMV IgG and development of neovascular AMD [35]. The presence of high anti-HCMV titers may indicate a subset of patients who harbor a greater total body burden of chronic HCMV infection, or who have experienced a recent, significant reactivation event. In either case, we hypothesize that the blood load of circulating HCMV-infected monocytes would be exceptionally high in this subset of patients. Upon recruitment to sites of drusen formation in patients who manifest the dry form of AMD, HCMV-infected monocytes would mature into tissue-resident macrophages with active virus replication, become polarized toward the pro-angiogenic M2 phenotype, and become a major source for production of a number of pro-angiogenic factors including VEGF that would amplify choroidal neovascularization associated with the wet form of AMD. We therefore believe that HCMV infection should be considered as a heretofore unrecognized risk factor for development of neovascular AMD. If true, subsets of patients who harbor a low virus load of HCMV would be predicted to experience decreased onset and progression of choroidal neovascularization, an occurrence that would impact their clinical outcome in terms of time of onset of visual loss and degree of visual loss. It is therefore possible that antiviral treatment might be effective in suppressing choroidal neovascularization associated with wet AMD in a fashion similar to that for suppression of allograft rejection in heart transplant recipients. Future studies will be oriented toward this investigation.

## Materials and Methods

### Ethics statement

All animal procedures were performed in strict accordance with the Association for Research in Vision and Ophthalmology Statement for the Use of Animals in Ophthalmic and Vision Research, and with the recommendations in the Guide for the Care and Use of Laboratory Animals of the National Institutes of Health. Animal research protocols were approved by the Institutional Animal Care and Use Committees of the University of Miami Miller School of Medicine (A3324-01) and Georgia State University (A3914-01). All laser treatments were performed under anesthesia (intramuscular administration of ketamine hydrochloride, xylazine, and adepromazine), and all efforts were made to minimize suffering.

### Mice

Adult female C57BL/6 mice were purchased from the National Institute of Aging (Bethesda, Maryland), and used throughout this investigation. Mice were allowed unrestricted access to food and water and maintained in alternating 12-hour light-dark cycles.

### Virus

Stocks of MCMV were prepared in mouse salivary glands as described previously [89]. Briefly, BALB/c mice (Taconic Farms, Germantown, New York) were infected intraperitoneally with 1×10^2^ to 1×10^3^ plaque-forming units (PFU) of the Smith strain of MCMV (American Type Culture Collection, Manassas, VA) contained within a 0.2-ml volume. Approximately 14 days later, the salivary glands were removed aseptically, homogenized (10% wt/vol) in Dulbecco's modified Eagle's tissue culture medium containing 10% fetal bovine serum (DMEM), clarified by centrifugation, and 0.25 ml aliquots of the supernatant stored in liquid N_2_. Virus stocks were titered on monolayers of mouse embryo fibroblasts (MEF) grown in DMEM. Fresh aliquots of MCMV stock were thawed and used for single experiments. UV-inactivated virus was prepared by exposure of aliquots of MCMV stock to ultraviolet radiation for 30 min to inactivate virus infectivity as determined by no detectable plaque formation on MEF monolayers 7 days after undiluted inoculation.

### Experimental plans

*Plan 1:* To evaluate CNV severity after acute or chronic infection with MCMV, four groups of mice (*n* = 10 mice per group) were injected intraperitoneally with 40 ul of a non-lethal dose of infectious MCMV (1.5×10^6^ plaque-forming units) or with an equivalent dose of UV-inactivated MCMV (controls). At 6 days (acute infection) and at 6 weeks and 12 weeks (chronic infection) after inoculation, the eyes of age-matched mice were subjected to bilateral laser treatment to induce CNV as described below. Mice were matched in age (10 months) at time of laser treatment. Four weeks later, the right eyes were collected for flat-mount analysis, and the left eyes were collected for histopathologic analysis.

*Plan 2:* To evaluate macrophages for their patterns of production of various pro-angiogenic factors during CNV at time of acute versus chronic MCMV infection, the eyes of groups of mice (*n* = 10 mice per group) were subjected to bilateral laser treatment at 6 days or at 12 weeks after intraperitoneal injection with infectious MCMV. The control group for this study consisted of groups of mice injected intraperitoneally with UV-inactivated virus. Mice were matched in age (10 months) at time of laser treatment for these animal groups. Four weeks after CNV induction, splenic macrophages were collected from all animals for quantitative RT-PCR assay analysis of several gene transcripts relevant to inflammation and/or neovascularization. Whole eyes, choroidal tissues, tissues from various organs (salivary glands, lung, spleen), and bone-marrow cells (CD34+ cells) were also collected from mice of the same animal groups and analyzed by standard plaque assay for detection of infectious virus or analyzed by PCR assay for detection of MCMV-specific DNA sequences.

*Plan 3*: To confirm mouse macrophages as a source for VEGF production following MCMV infection, monolayers of the IC-21 mouse macrophage cell line (American Type Culture Collection, Manassas, VA, USA) [41] were inoculated either with MCMV (moi = 2.5), UV-inactivated MCMV, maintenance medium only, or maintenance medium containing lipopolysaccharide (LPS) (100 ng/ml). All cells were harvested at 24 or 48 hrs postinfection and subjected to quantitative real time RT-PCR assay for quantification of VEGF mRNA and TNF-α mRNA, standard ELISA for quantification of VEGF protein production, or immunostaining for detection and pattern of VEGF production.

*Plan 4:* To determine the effect of antiviral treatment on production of VEGF mRNA and TNF-α mRNA by splenic macrophages at time of chronic MCMV infection, groups of mice (*n* = 10 mice per group) were injected intraperitoneally with 40 ul of a non-lethal dose of infectious MCMV (1.5×10^6^ plaque-forming units) or maintenance medium (mock infected). At 12 weeks after inoculation, MCMV-infected or mock-infected mice were treated intraperitoneally with ganciclovir for 7 days at a dose of 40 mg/kg/day, a dose that reflects the relative decreased sensitivity of MCMV to ganciclovir when compared with the sensitivity of HCMV to ganciclovir [43]. Untreated control MCMV-infected or mock-infected mice received daily intraperitoneal injections of phosphate-buffered saline for 7 days. Following the 7-day regimen of ganciclovir or phosphate-buffered saline treatment, splenic macrophages were collected from ganciclovir-treated and untreated chronically infected mice and compared by quantitiative real time RT-PCR assay for levels of VEGF mRNA production. To determine the effect of antiviral treatment on production of VEGF mRNA and TNF-α mRNA by mouse macrophages during acute MCMV infection, monolayers of IC-21 mouse macrophages were inoculated either with MCMV (moi = 2.5) or mock-infected with maintenance medium. At 1-hr postinfection, MCMV-infected and mock-infected monolayers were treated either with 15, 30, or 60 uM of ganciclovir or treated with phosphate-buffered saline (control). At 24 hr postinfection, all monolayers were harvested and subjected to quantitative RT-PCR assay for quantification and comparison of levels of VEGF mRNA production.

### Mouse model of laser-induced CNV and morphometric analysis of severity

At 6 days, 6 weeks, or 12 weeks after injection with infectious or UV-inactivated MCMV, diode red laser was used to create choroidal thermal burns bilaterally and induce experimental CNV as described previously [39]. Four weeks after laser application, mice were euthanized, and subjected to histopathologic analysis as well as flat-mount analysis of surface area, vascularity, and cell density of CNV. All images were digitally acquired (Axiovision, Zeiss) and recompiled (Photoshop version 6.0; Adobe, San Jose, California). Surface area of CNV lesions was determined by using either fluorescein-isothiocyanate (FITC)-dextran (Sigma, St. Louis, Missouri) fluorescence or propidium iodide (PI, Sigma) fluorescence, and outlining the margins of the lesion with a computer analysis software (Photoshop 6.0). The area in pixels was normalized by dividing the average of the optic disc measured in 10 independent eyes. Five eyes were examined 4 weeks after laser treatment to determine the average spot size (0.48 disc areas). A CNV was determined to be present if the surface area of an individual lesion was greater than 0.50 disc areas.

### Histopathologic analysis

Four weeks after bilateral laser treatment of groups of mice infected systemically with MCMV for 6 days, 6 weeks, or 12 weeks, left eyes were carefully removed from all animals following euthanasia, fixed in 10% buffered formalin, paraffin embedded, sectioned with hematoxylin and eosin, and examined by light microscopy for detection and quantification of areas of CNV.

### Macrophage collection and enrichment

Following removal of spleens under sterile conditions from euthanized mice, a Spectra/Mesh macroporus 210 µm filter (Spectrum Laboratories, Inc., Los Angeles, California) was used to obtain splenic macrophages after maceration of individual spleens in a Hanks balance salt solution (HBSS) medium containing 1 M HEPES, 1 M NaAZ, and fetal bovine serum. ACK buffer was added to the spleen suspension to lyse red blood cells. The remaining cells were centrifuged and resuspended in HBSS medium containing rat anti-mouse F4/80 conjugated with PE (Caltag, Burlingame, California). Splenic macrophages were then purified by magnetic column separation using MACS Anti-PE Microbeads (Miltenyi, Auburn, California) as specified by manufacturer's instructions.

### Whole bone marrow cell collection and enrichment

At the time of euthanasia and under sterile conditions, tibias and femurs were dissected and bone marrow was extracted by slowly flushing the dyaphyseal channel with HBSS medium using a 27-gauge needle. Bone marrow was homogenized, filtered, centrifuged, and resuspended in HBBS medium. Red blood cells were lysed with ACK buffer, and the remaining cells were incubated with rat anti-mouse CD34 conjugated with PE (BD Biosciences, Pharmingen, San Diego, California). CD34+ vascular precursor cells were then purified by magnetic column separation using MACS Anti-PE Microbeads (Miltenyi) as specified by manufacturer's instructions.

### Detection of infectious MCMV

Whole eyes collected from mice at 30 days after laser-induced CNV were frozen individually at −80°C. At time of quantitative plaque assay, eyes were thawed, homogenized individually in 1.0 ml of cold Dulbecco's modified Eagle's medium (DMEM) containing 10% fetal bovine serum, and clarified by centrifugation. Ten-fold dilutions of the resulting supernatants were titered in duplicate onto monolayers of MEF contained within 6-well plates, allowed to absorb for 1 hour at 37°C, overlaid with methylcellulose containing DMEM, and incubated for 5 or 6 days at 37°C in a humidified CO_2_ atmosphere. Monolayers were screened daily for 7 days using an inverted light microscope for detection of plaques of MCMV-induced cytopathology.

### PCR assay for MCMV-specific DNA

DNA was extracted from whole eyes, salivary glands, lungs, bone marrow, spleens, and isolated macrophages collected from euthanized mice using the QIAamp Tissue kit (QIAGEN GmbH, Valencia, California) according to manufacturer's instructions and subjected to PCR assay to detect MCMV-specific DNA using primers for immediate early 1 (IE1) and glycoprotein H (gH) genes. The primers used were kindly provided by Dr. Daniel D. Sedmak, Ohio State University College of Medicine, Columbus, Ohio. The primer pair for MCMV IE1 gene was 5′-TAGCCAATG ATATCTTCGAGCG-3′ and 3′-ATCTGGTGCTCCTCAGATCAGCTAA-5′, and the primer pair for MCMV gH gene was 5′-TTCAGTTCAACTCGAA-3′ and 3′-GGGAAGAAGTACTCGACCGG-5′. PCR amplification of β-actin was performed as an internal control. Actin primers consisted of 5′-ATTGTGATGGACTCCGGTGA-3′ and 3′-AGCTCATAGCTCTTCTCCAG-5′. DNA extracted from tissue homogenates was eluted in 100 µl of distilled water, and stored at −20 C until analysis. DNA was amplified in a total volume of 25 µl with 200 nM of each primer and 1.0 U of Taq DNA polymerase (Gibco BRL) added in 2.5 µl of a PCR buffer (50 mM KCL, 20 mM Tris-HCl [pH 8.4], and 1.5 mM MgCl_2_). PCR assays were performed on a Perkin Elmer 9600 thermocycler (PE Applied Biosystems). PCR assay conditions consisted of an initial denaturation step of 4 min at 94 C, followed by 35 cycles, with 1 cycle consisting of 30 sec at 94 C, 30 sec at 53 C, and 30 sec 72 C. Amplification products were separated by electrophoresis through 1% agarose gels, and stained with ethidium bromide for visualization.

### Quantitative real-time RT-PCR assay

Total RNA was extracted from whole bone marrow cells (CD34+ cells), splenic macrophages, or MCMV-infected IC-21 mouse macrophage monolayers using Tri-Reagent and prepared for quantitative RT-PCR reactions as described previously [90]. Real time RT-PCR assay was used to quantify several cellular transcripts of interest that included mouse tumor necrosis factor-alpha (TNF-α), matrix metalloproteinase-9 (MMP-9), vascular endothelial growth factor (VEGF), VEGF receptor 1 (VEGFR1), VEGF receptor 2 (VEGFR2), platelet-derived growth factor-beta (PDGF-β), cyclooxygenase (COX-2), and inducible nitric oxide synthase (iNOS). Real time RT-PCR assays were performed for TNF-α, VEGFR1, VEGFR2, and COX-2 mouse transcripts using commercially available kits (Perkin Elmer Applied Biosciences). The primer pair for real time RT-PCR assay of mouse PDGF-β mRNA was 5′-AAGCACACGCATGACAAG-3′ and 3′-GGGGCAATACAGCAAATAC-5′; for VEGF mRNA was 5′- CGAAACCATGAACTTTCTGC-3′ and 3′-CCTCAGTGGGCACACACTCC-5′; for MMP-9 mRNA was 5′-CAGGATAAACTGTATGGCTTCTGC-3′ and 3′- GCCGAGTTGCCCCCA-5′; and for iNOS mRNA was 5′-TGACGCCAAACATGACTTCAG-3′ and 3′-GCCATCGGGCATCTGGTA. Transcripts of these molecules were normalized to 18S ribosomal RNA transcripts via standard curves generated using serially diluted samples of mRNA (0.001–100 ng). Real time RT-PCR assays were performed in duplicate with quantitative values determined for each molecule as the ratio of the mean values for a specific mRNA versus 18S mRNA. Median values for each molecule were calculated and normalized to samples obtained from sham-inoculated control animals (100%).

### Immunostaining for detection of VEGF

MCMV-infected and mock-infected monolayers of IC-21 mouse macrophages grown on 6-well chamber slides were harvested at 24 and 48 hr postinfection, fixed in cold ethanol, dried, and reacted with 5% normal goat serum containing 0.2% Triton X-100. Following three washings in phosphate-buffered saline, slides were incubated for 1 hr with either rabbit anti-mouse VEGF IgG (1∶100 dilution) (Santa Cruz Biotechnology, Santa Cruz, CA) or normal rabbit IgG (1∶100 dilution (Santa Cruz Biotechnology, Santa Cruz, CA), washed three times with phosphate-buffered saline, and reacted with biotinylated anti-rabbit IgG secondary antibody using the Rabbit ABC Staining system (Santa Cruz Biotechnology, Santa Cruz, CA). Chamber slides were mounted on standard microscope slides and cell nuclei were counterstained using Vectashield Mounting Medium containing DAPI (Vector Laboratories, Burlingame, CA). All slides were examined and photographed using a Nikon Eclipse 50i microscope equipped with an X-Cite Series 120 Epi-fl illuminator.

### Statistical analysis

Morphometric data for individual lesions in each eye were averaged to provide one value per eye. Mean and standard deviation values for each group was calculated and *p* values were determined using student *t*-test and one-way analysis of variance+Dunnett's multiple comparison post-hoc test (GraphPad Prism 4.0, San Diego, CA). Values of *p*≤0.05 were considered statistically significant for all forms of statistical analysis used.
